# A method for imaging single molecules at the plasma membrane of live cells within tissue slices

**DOI:** 10.1085/jgp.202012657

**Published:** 2020-12-16

**Authors:** Gregory I. Mashanov, Tatiana A. Nenasheva, Tatiana Mashanova, Catherine Maclachlan, Nigel J.M. Birdsall, Justin E. Molloy

**Affiliations:** 1 The Francis Crick Institute, London, UK; 2 Russian Academy of Science, Koltzov Institute of Developmental Biology, Moscow, Russia; 3 Village Vet Potters Bar, Potters Bar, UK

## Abstract

Recent advances in light microscopy allow individual biological macromolecules to be visualized in the plasma membrane and cytosol of live cells with nanometer precision and ∼10-ms time resolution. This allows new discoveries to be made because the location and kinetics of molecular interactions can be directly observed in situ without the inherent averaging of bulk measurements. To date, the majority of single-molecule imaging studies have been performed in either unicellular organisms or cultured, and often chemically fixed, mammalian cell lines. However, primary cell cultures and cell lines derived from multi-cellular organisms might exhibit different properties from cells in their native tissue environment, in particular regarding the structure and organization of the plasma membrane. Here, we describe a simple approach to image, localize, and track single fluorescently tagged membrane proteins in freshly prepared live tissue slices and demonstrate how this method can give information about the movement and localization of a G protein–coupled receptor in cardiac tissue slices. In principle, this experimental approach can be used to image the dynamics of single molecules at the plasma membrane of many different soft tissue samples and may be combined with other experimental techniques.

## Teaser

Mashanov et al. present a method to image and track individual fluorescently labeled molecules in live cardiac tissue slices using total internal reflection fluorescence (TIRF) microscopy. They show that the movement and interaction of G protein–coupled receptors can be observed in real time.

## Introduction

Over the past 25 yr, light microscopy has undergone a radical transformation due to development of sensitive cameras, solid-state lasers, and fast computers. So, it is now relatively straightforward to visualize individual fluorophores in aqueous solution and in living cells. Early single-molecule imaging studies were made using surface-immobilized, biological molecules in vitro (Funatsu et al., 1995; Vale et al., 1996; Noji et al., 1997; Lu et al., 1998). Later, the same imaging approaches were applied to living cells (Sako et al., 2000; Harms et al., 2001; Iino et al., 2001; Mashanov et al., 2003) with the broad aim of learning more about biomolecular interactions in vivo by direct observation. In the current study, we have advanced this approach by developing a relatively simple method to study individual molecules in freshly explanted live tissue slices. We have visualized individual M_2_ muscarinic acetylcholine receptors moving within the plasma membrane of mouse and zebrafish cardiac tissue slices, and we find that membrane properties are radically different from primary cell cultures and cell lines. The physico-chemical properties of lipid membranes of unicellular organisms (prokaryotes and primitive eukaryotes) are critical to cell viability, cell maintenance, and cell signaling, while in multicellular organisms, cell–cell contacts and interactions also become critical to the development, viability and integrity of the organism. Direct observation of integral membrane proteins within the plasma membrane of living cells and of cells embedded within live tissue samples opens new avenues for research.

To measure the kinetics of a molecular process, data must be sampled faster than the relaxation rate of the underlying biochemical or biophysical process. Kinetics of protein–lipid interactions vary over more than seven orders of magnitude from microseconds to tens of seconds. So, while some processes can be observed via video imaging methods (often called “direct observation”), others require more sophisticated optical correlation techniques. In this paper, we focus on direct observation of single molecules by video microscopy, which is currently limited to a temporal range from around 10 ms out to several tens of seconds (bandwidth of ∼0.01–50 Hz). This is sufficient to fully characterize the lateral diffusion of proteins and their collisional interactions in lipid bilayers but, for instance, is insufficient to directly observe their rotational diffusion. To visualize single fluorophores within a live biological specimen, fluorophores must be bright, sparse, and tolerant to photobleaching. The microscope must have diffraction-limited performance with good mechanical stability and be equipped with a camera system that is sensitive, fast, and low-noise. To put some approximate numbers on things, a cubic micron of cytosol contains >10^8^ biological molecules, many of which autofluoresce or produce Raman scattering. So, synthetic fluorophores (such as rhodamine) and fluorescent proteins (such as GFP) must be used as molecular probes. Their intense fluorescence at long wavelengths (λ > 450 nm) means they appear as bright spots of light above the diffuse cytosolic autofluorescence. A green fluorophore (λ = 550 nm) viewed in aqueous solution (refractive index = 1.3, allowing a maximum numerical aperture [NA] = 1.3) appears as a circular spot, ∼250 nm in diameter, in the x-y plane (given by Abbe’s theory, 0.5λ/NA) and ∼600 nm along the focal axis (2λ/NA^2^). So, in order to visualize two fluorophores as distinct objects, they must be separated by ∼250 nm (the Raleigh criterion). To achieve the requisite fluorophore separation, either labeled molecules must be naturally present at low concentration (<1 nM; i.e., <1 molecule/μm^3^), or only a fraction (<0.1%) of the molecules should be tagged, or fluorophores that blink on and off, with a low duty-cycle ratio, can be used such that they spend perhaps 0.1% of the time in a bright fluorescent on state and 99.9% of the time in a dark off state.

TIRF microscopy (TIRFM) offers a great improvement over standard episcopic fluorescence excitation because the illuminating light beam no longer penetrates the entire specimen, and background autofluorescence is greatly reduced. To produce TIRF excitation, a focused laser beam enters the extreme edge of the objective lens (NA must be >1.4) so it is then incident to the specimen plane at greater than the critical angle (>63°), leading to total internal reflection at the interface between the glass coverslip and the (lower refractive index) aqueous medium of the biological specimen. This illumination method gives rise to an evanescent field that penetrates the specimen to a narrow depth (1/e = 100 nm; Axelrod, 1989). This means the excitation point-spread function along the z axis is dramatically reduced compared with standard episcopic illumination and background fluorescence from out-of-focus fluorophores, and cell autofluorescence is dramatically reduced (Funatsu et al., 1995; Sako et al., 2000). By careful optical design (Mashanov et al., 2003), the evanescent field can be made to propagate across the object plane, giving a wide-field image of the specimen (100 × 100 µm^2^ area). Given the planar nature of the plasma membrane, TIRFM is ideally suited to studies of membrane proteins and protein–lipid interactions. Other approaches to reduce background fluorescence include confocal microscopy, light-sheet microscopy, and laser-based, oblique-angle, illumination microscopy (Kues et al., 2001; Tokunaga et al., 2008; Reyes-Lamothe et al., 2010), which have various advantages for single-molecule imaging deeper inside the cell. Here, we focus on use of TIRFM because it is so well-suited to studies of membrane biology.

To make video recordings of individual fluorophores, the camera system should have high signal-to-noise ratio, i.e., high photon collection efficiency (product of detector quantum efficiency and pixel fill factor) compared with the sum of all noise sources (electrical readout noise, digitization noise, and detector noise). To track individual molecules in aqueous solution, at room temperature, the exposure time (shutter speed) must be long enough to collect sufficient light required to localize the fluorophore but brief enough that the image is not blurred by diffusive motion. Fluorophore brightness (emission rate) increases linearly with laser power until the emission is limited by the relaxation time of the excited state, called “saturation,” which approaches ∼10^7^ photons per second. However, at saturating laser excitation power, the average observation time before photobleaching is often just a few milliseconds. So, there is an important trade-off between how intensely the sample is illuminated, which determines the fastest possible sample rate (shortest times) and the duration of the observation (longest times), called the “observation time window,” which is equivalent to bandwidth in the frequency domain. From a practical standpoint, excitation intensity (i.e., laser power) is adjusted to give an emission rate and fluorophore bleaching rate that optimize the observation time window for a specific measurement (for an example of a more rigorous treatment, see Michalet, 2010). So, for instance, a high laser power and fast frame rate are used to study fast events, and a low laser power and slow frame rate are used to study slower events. If we want to study the diffusive motion of a protein, then the required number of frames per second (fps) can be approximated by first assuming the distance moved between frames should be around 300 nm so that individual fluorophore paths can be tracked unambiguously when molecules are present at a reasonable surface density (e.g., 0.25 µm^−2^), allowing large datasets to be accumulated. Then, knowing the lateral diffusion coefficient (D_lat_) of a typical protein (e.g., molecular weight, 40 kD) in cytoplasm is ≈20 µm^2^ ⋅ s^−1^ and in lipid membrane is ≈0.2 µm^2^ ⋅ s^−1^ (Saffman and Delbrück, 1975), we can calculate the minimum required frame rate from the inequality (2D_lat_/fps)^0.5^ ≤ 300 nm, giving ≥500 fps for molecules in cytoplasm and ≥5 fps for molecules in membranes. With this knowledge, laser power and camera frame rate can be adjusted to suit the task, and in fact, they can be systematically varied while viewing the same specimen in order to sample different observation time windows.

To date, most live-cell, single-molecule imaging studies have been performed using either unicellular organisms (yeast and bacteria) or cultured adherent mammalian cells, either from stable cell lines or primary cell culture (Ueda et al., 2001; Mashanov et al., 2004, 2010; Hern et al., 2010; Achimovich et al., 2019). It is generally assumed that adherent, isolated cells are reliable models for cells within their native tissue. However, this is not always the case: for example, it was found that M_2_ muscarinic receptors move fourfold faster in intact mouse hearts (D_lat_ ≈ 0.6 µm^2^ ⋅ s^−1^) compared with isolated primary cardiomyocytes (D_lat_ ≈ 0.15 µm^2^ ⋅ s^−1^; Nenasheva et al., 2013), and since G protein–coupled M_2_ muscarinic receptor signaling is critically dependent on diffusive interactions with inwardly rectifying potassium channels (Reuveny et al., 1994; Huang et al., 1995), signaling kinetics may be quite different in intact muscle from model cell systems. To understand how biological macromolecules function within cells, tissues, and intact living organisms, it is necessary to develop techniques that span different experimental paradigms. To address this, we present a simple method of sample preparation and a TIRF imaging approach that allows single-molecule imaging and tracking within cultured adherent cells and also ex vivo tissue slices. We demonstrate the approach using cardiac tissue from mouse and zebrafish (Hsieh and Liao, 2002), and show how super-resolution, single-molecule localization informs us about molecular dynamics and can also be used to obtain ultrastructural information from heart muscle.

## Materials and methods

All chemicals were obtained from Sigma-Aldrich unless stated otherwise.

### Tissue preparation

**Figure 1.**
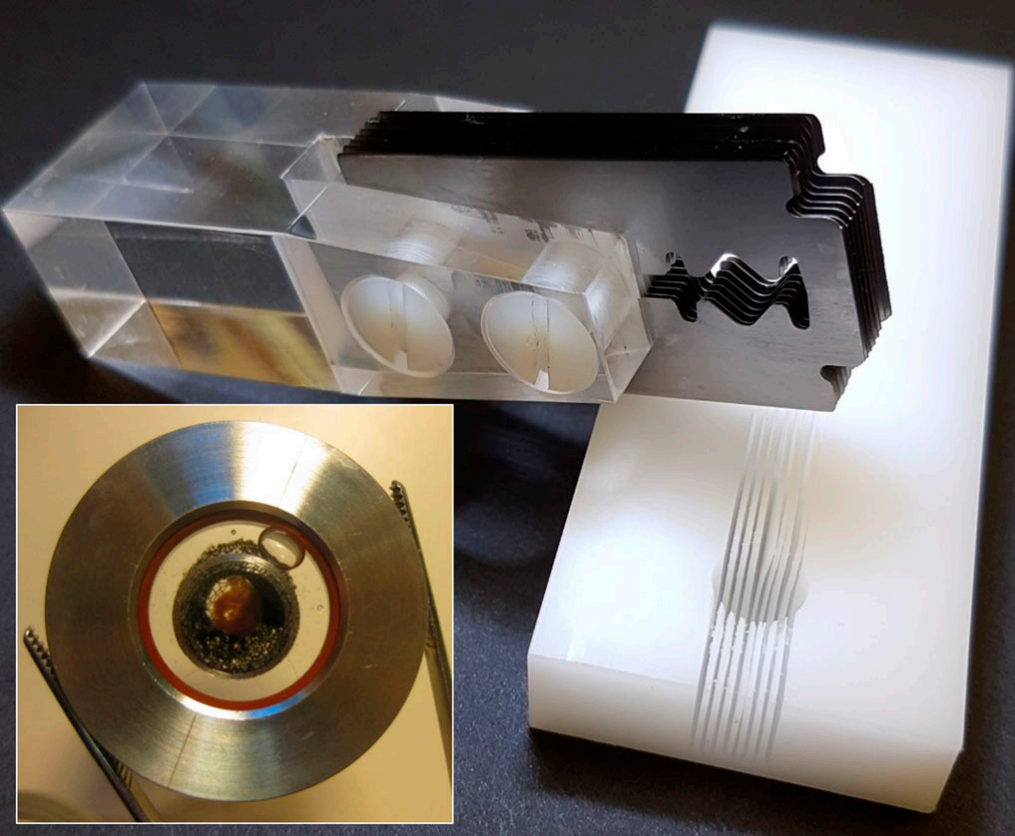
**Preparation of ex vivo tissue slices.** The matrix cutting block (right) has an 8-mm-diameter hemispherical cavity with eight slots, 0.2 mm wide (on 1-mm centers). An isolated heart is placed into the cavity, and a set of eight razor blades (0.1-mm thick, spaced 1 mm apart, screwed to a Plexiglas handle; top left), is inserted into matching slots in the cutting block so the heart can be cut into multiple sections in one stroke. The assembled imaging chamber (inset) contains a cardiac tissue slice on the lower coverslip, held in place by a nylon mesh stretched across a stainless steel tambour.

It is important to shorten the time between the extraction of an organ and its imaging in order to minimize the effects of disruption to blood supply and subsequent hypoxia. Freshly extracted mouse heart was placed in warm PBS (pH 7.2, 35°C; Thermo Fisher Scientific) solution supplemented with 10 U ⋅ ml^−1^ heparin and 100 U ⋅ ml^−1^ penicillin-streptomycin for 5 min. After washing, the heart was moved into ice-cold “relaxing PBS” (consisting of PBS supplemented with 1 mM EDTA, 2.5 mM KOH, and 3 mM MgCl_2_) for 5 min. The washed heart was then placed into the 8-mm-diameter cavity of a custom-made plastic matrix cutting block (Fig. 1) oriented with the long axis of the heart ventricles either parallel (sagittal sectioning) or orthogonal (transverse sectioning) to the cutting slots. A stack of eight standard razor blades (thickness ∼0.1 mm, spaced 1 mm apart) screwed to a Plexiglas handle was engaged into corresponding slots in the matrix cutting block, and the heart was then sectioned in a single stroke using a smooth sliding motion of the multi-blade assembly to produce a series of 1-mm-thick slices (CAD drawing files and STL files for 3-D printing are freely available on request). The order of the slices was noted to keep track of the heart anatomy. For the zebrafish experiments, a smaller cutting block with a 2-mm-diameter cavity and a single cutting slot was used (not shown) to bisect the heart along the axis of its single ventricle.

### High-affinity fluorescent labeling of muscarinic receptors

The prepared tissue slices were placed in relaxing PBS solution supplemented with 10 nM of Cy3B-telenzepine (Hern et al., 2010; Nenasheva et al., 2013) at 4°C for an hour in the dark. This procedure labels >95% of M_2_ muscarinic receptors with the tight-binding fluorescent ligand. In all cases, heart slices were washed several times in fresh relaxing PBS solution to remove any unbound Cy3B-telenzepine. Cy3B-telenzepine bound to M_2_ receptors is not removed by washing because of its extremely slow dissociation rate. The prepared slice was moved to a fibronectin-coated circular coverslip (no. 1, 25 mm diameter) inserted into a custom-made imaging chamber (Fig. 1). The tissue slice was pressed firmly against the coverslip using a fine nylon mesh grid (0.5 × 0.5 mm^2^) stretched in a stainless steel ring. The chamber was sealed and filled with Hanks’ balanced salt solution supplemented with 20 mM HEPES (pH 7.4). The imaging was performed at 23°C or 37°C.

### Scanning confocal microscopy

Confocal microscopy was performed at 23°C using a Leica SP5-II confocal microscope (Leica Microsystems) equipped with a GaAsP hybrid photomultiplier detector, 561 nm laser excitation source, and 20×, NA 1.0, water-dipping objective lens, giving, 200 nm/pixel in the x-y plane and using 1-µm z-steps to acquire specimen volume sections.

### Critical-point drying and scanning EM

Slices were fixed in 0.1 M phosphate buffer (PB; 2.5% glutaraldehyde and 4% formaldehyde, pH 7.4) for 1 h at room temperature, then washed in 0.1 M PB (three times for 5 min) and incubated in 1% reduced osmium (1% osmium tetroxide and 1.5% potassium ferricyanide) for 1 h at 4°C. Slices were then washed in 0.1 M PB (three times for 5 min) followed by double-distilled water (three times for 5 min) and dehydrated in a graded ethanol series (20%, 50%, 70%, 90%, 100%, 100%; 20 min each) and dried using a critical-point dryer (EM CPD300; Leica Microsystems). Slices were mounted onto scanning electron microscope stubs (10-002012-100; Labtech) using silver paint (AGG3691; Agar Scientific) and coated in 2 nm of platinum (Q150RS; Quorum). Images were acquired using an Everhardt-Thornley secondary electron detector in a scanning electron microscope (Quanta FEG 250; Thermo Fisher Scientific) at 6 keV, 2 nm spot size, 5 µs dwell time, and 15 mm working distance.

**Figure 2.**
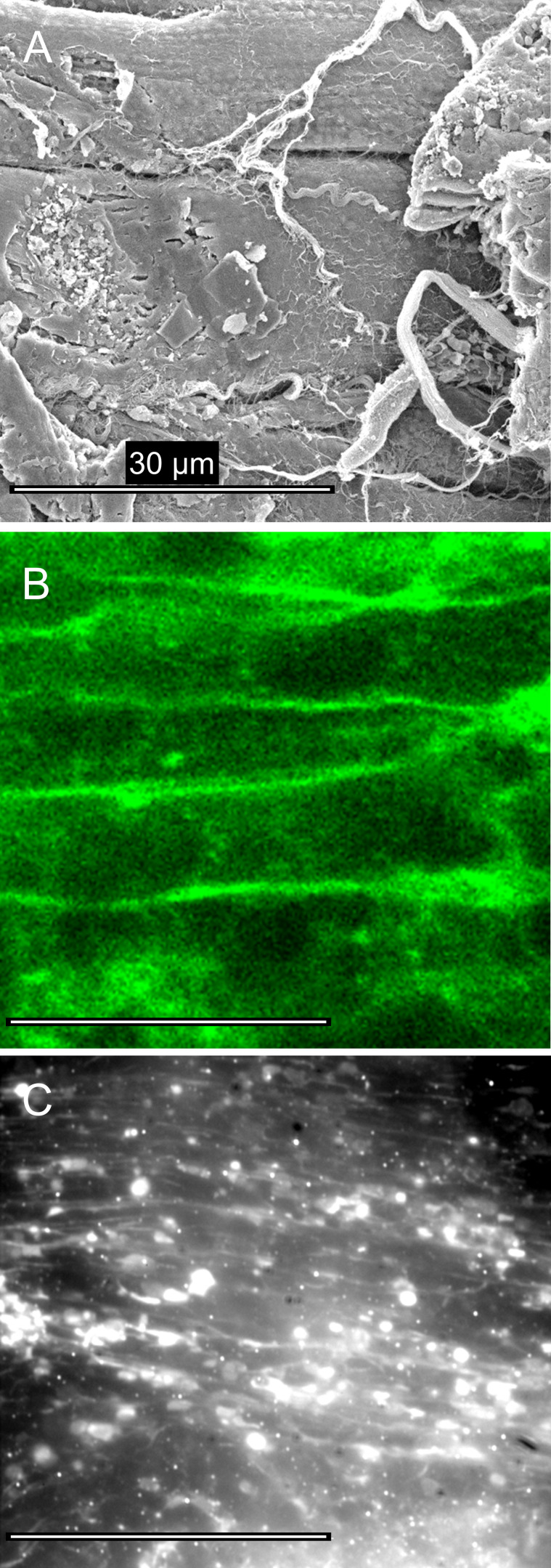
**Tissue morphology after the preparation of ex vivo heart slices.** **(A)** Scanning electron micrograph of a critical-point dried cardiac tissue section from adult mouse shows regions of damage where myocytes are cut obliquely (right side) and other regions where muscle cell plasma membrane is relatively intact (see text for details). **(B)** Scanning confocal optical microscopy, using the membrane dye (DeepRed Cellmask, 10 µg ⋅ ml^−1^, for 10 min; Invitrogen) shows that the cellular structures in optical sections taken 1 µm beyond the cut surface were well-preserved. **(C)** Tissue sections, labeled with Cy3-telenzepine, viewed by TIRFM, were heterogeneous. Some regions were well-preserved while others showed significant cell damage. Bright circular regions occur where the plasma membrane is closely opposed to the coverslip surface and enters the evanescent field.

Like all other physical sectioning methods, cell damage occurs at the cut interface. We visualized the tissue surface by critical-point drying and scanning EM and by scanning confocal microscopy (Fig. 2, A and B). Muscle cells were best preserved when sectioned along the fiber axis, but regions of cell damage were evident (Fig. 2 A). Tissue slices continued to show rhythmic contractions following the sectioning procedure. TIRF imaging results reported in the current study relate to sample regions that showed the best cell preservation (Fig. 2 C). For studies in which cell integrity is absolutely critical to the experiment, combining TIRF and confocal imaging in the same specimen would be advantageous.

### TIRF imaging conditions

A custom-built TIRFM microscope was used, as described previously (Mashanov et al., 2003). Briefly, the beam from a 100 mW 556-nm laser (MGL-556–100; Suwtech) was expanded using a Galilean beam expander and focused at the back focal plane of a high NA, oil-immersion, objective lens (AlphaPlan, 100×, NA 1.45; Carl Zeiss) using a small aluminum-coated mirror (3 mm diameter; Comar Optics) placed at the edge of the back aperture of the objective lens. The average laser intensity at the specimen plane was adjusted to 20–50 µW ⋅ µm^−2^, and the incident laser beam angle was adjusted to ∼63° to create the evanescent field at the glass–aqueous medium interface. A second small mirror was placed at the opposite edge of the objective lens back aperture to remove the returning (internally reflected) laser beam from the microscope, and a narrow band-pass emission filter FF01-585/29 (Semrock) was used to block the scattered 556 nm laser light and other unwanted light. An electron multiplying charge-coupled device camera (iXon897BV; Andor) captured full-frame (512 × 512 pixels) video sequences at 20 fps, and using the Andor camera “cropped-mode” (e.g., 512 × 100 pixels) at 100 fps. Data were stored on a computer hard drive for later analysis.

### Comparing mobility on basal and apical cell membranes

We checked if the diffusion of labeled muscarinic receptors differs on basal (adjacent to the coverslip) and apical cell membranes by labeling an atrial cardiomyocyte line, HL-1, with Cy3B-telenzepine (cells were prepared and cultured as described previously; Mashanov et al., 2010). Fluorescent molecules were imaged on the apical plasma membrane by decreasing the incident laser beam angle below the critical angle in order to obliquely illuminate (Tokunaga et al., 2008) the apical cell surface at a distance 2–3 µm above coverslip surface. Single-molecule tracking gave an average D_lat_ = 0.13 µm^2^ ⋅ s^−1^ (2,151 trajectories measured in 6 cells) that was similar to D_lat_ = 0.12 µm^2^ ⋅ s^−1^ (1,787 trajectories in 3 cells) measured at the basal surface (Video 1). We conclude there is little difference in M_2_ receptor mobility on the upper and surface-adhered plasma membranes.

**Video 1.** **Comparison of receptor mobility at the basal surface and apical, upper surface in a live, cultured, cardiac cell line (HL1 cell).** Basal surface imaging performed in TIRF image mode, and upper surface imaging performed using oblique angle laser illumination.

### Video data analysis

Video image sequences were analyzed using GMimPro software (https://www.mashanov.uk; Mashanov, 2007), which employs an automatic single particle tracking algorithm (described previously in Mashanov and Molloy, 2007) to detect and track individual fluorophores. The position of each fluorescent spot was localized with sub-pixel resolution using a Gaussian fitting method, which gave a localization resolution of ∼10 nm at each frame. Individual particle trajectories were then output as a table of floating-point x-y coordinates measured at each video frame (i.e., each time point) for every fluorophore detected in the sample. From the tabulated data, the mean squared displacement (MSD) of each fluorophore was computed over all possible time intervals (dT; e.g., 1, 2, 3…*n* frames). MSD versus dT plots were then generated to examine the particle motion; a linear relationship is expected for simple Brownian motion, but curvature indicates anomalous diffusion. We analyzed trajectories where single fluorescent spots were tracked for at least eight consecutive frames with a maximum displacement of ≤0.7 µm between frames (Mashanov and Molloy, 2007) to avoid erroneous connection of trajectories belonging to different molecules. If the images of individual fluorophores overlap due to molecules moving close to each other, one trajectory terminates and a new trajectory is generated when the molecules move apart.

### Recombining single-fluorophore videos to show ensemble behavior

**Figure S1.**
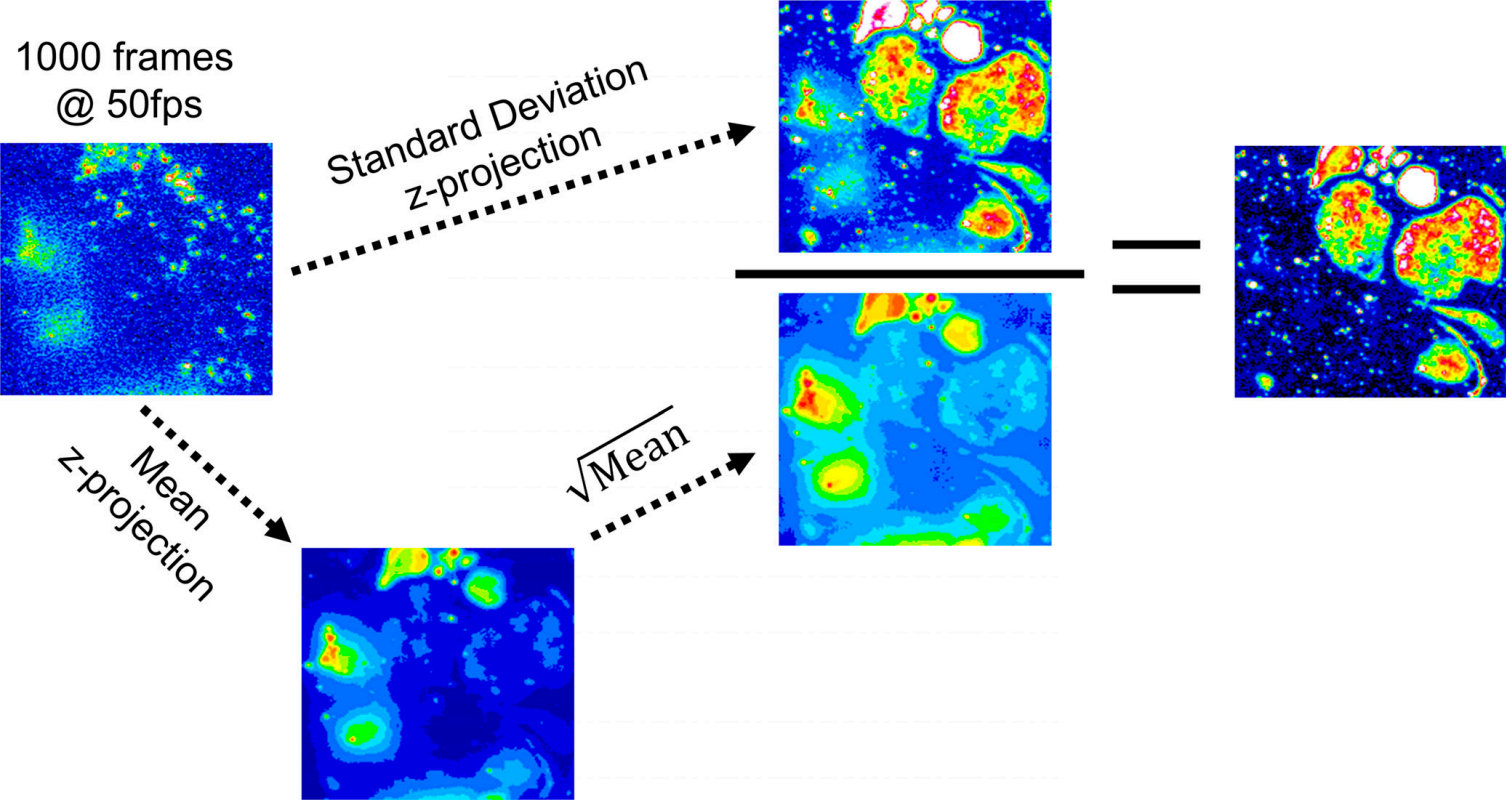
**Construction of the normalized SD z-projection maps.** The original video record is represented by the first frame in the image stack (top left). The image stack is z-projected as a single frame of the same pixel dimensions corresponding to the SD in pixel intensity (the “numerator,” top center) or the mean of pixel values (bottom left). The intensity variations are due to shot-noise scale with the square-root of mean intensity (the “denominator,” bottom center). The resulting projection is the “normalized” SD (right). Note that bright but diffuse regions on the left side of the original image disappear, whereas regions where fluorescent molecules move rapidly at the cell membrane (top right regions of the image) have large fluctuations in pixel intensity that are greatly enhanced in the normalized SD z-projection.

In most fluorescence imaging studies, cells are brightly labeled and visualized for relatively long exposure times (>0.5 s) in order to reveal the details of cellular structures. In live-cell imaging, time-lapse video microscopy is often used to reveal intra-cellular movements. The approach used here is to apply high-speed “movie-mode” imaging of sparsely labeled cells where the motion of individual molecules can be studied. Data can be visualized as a “z-projection” (where the z-dimension is time) whereby each pixel value represents a statistical descriptor of pixel intensity over time. Commonly used z-projections are median, average, maximum, or SD in pixel value measured over the duration of the video time series. The SD in pixel intensity should scale with the square root of mean intensity (shot-noise limit). However, for sparsely labeled live-cell video recordings’ fluctuations in pixel intensity are dominated by fluorophore motion and/or appearance and disappearance of individual fluorophores at a given pixel location. In this situation, the normalized SD projection,

$$\sqrt{\left(\frac{\sum^{\text{​}}{\left(\bar{I}-I_{t}\right)}^{2}}{\sum^{\text{​}}I_{t}}\right)},$$

where $\sum^{\text{​}}{\left(\bar{I}-I_{t}\right)}^{2}$ is pixel intensity variance and $\sum^{\text{​}}I_{t}$ is summed intensity, corrects for the expected shot-noise contribution and enhances contrast in regions of high-intensity fluctuation due (mainly) to fluorophore movement (Fig. S1).

### Super-resolution images by single-molecule “stop-motion” analysis

To create a super-resolution image of the tissue slice, videos of 30–60 s duration were recorded, i.e., ∼1,000 video frames, typically comprising >100,000 single-fluorophore spots. To analyze these data, fluorescent spots were first identified by a spot-finding algorithm (as above), and immobile spots were manually selected and used as fiducial markers so video data could be drift-corrected (usually <5–10 nm ⋅ s^−1^). After drift correction, each fluorescent spot was centroided, and its floating-point, x-y coordinate pair (resolved with ∼10 nm precision), was replotted onto a new, up-sampled (“zoomed”), image frame to produce a super-resolution map of the trajectories. In these representations, if the original camera image was 512 × 512 pixels, with 100 nm per pixel calibration, the resulting super-resolution image might be an image of 5,120 × 5,120 pixels at 10 nm per pixel. This type of stop-motion analysis allows us to map cellular ultrastructures such as the cytoskeleton (Baboolal et al., 2016), sub-cellular organelles (Mashanov et al., 2010), or lipid domains (Dietrich et al., 2002).

### Single-molecule mobility maps

We created a map of protein mobility by pseudocolor-coding each fluorophore track based on the initial gradient of its MSD versus dT relation. This approach is similar, but not identical, to single-particle velocimetry. At positions where trajectories overlap, pixel intensity was the average of all tracks passing through that location. For display purposes, pseudocolor-coding was used in order to give a visual heat map of fluorophore mobility across the field of view.

### Online supplemental material

Fig. S1 shows normalized SD z-projection maps that reveal regions of intensity fluctuation that exceed the expected shot-noise contribution. Video 1 is a comparison of receptor mobility at the basal surface (TIRF imaging mode) and apical, upper surface (using oblique angle laser illumination) in a live, cultured, cardiac cell line (HL1 cell). Video 2 is a side-by side comparison of receptor density and mobility in an isolated cardiomyocyte and in a heart slice from the ventricle of a mouse of the same age (0.5× real time). Video 3 shows a slice of the zebrafish adult heart is still beating after 1 h labeling at 4°C. Video 4 shows M_2_ receptor mobility in the adult zebrafish heart slice. Video 5 shows that tracking individual muscarinic receptors on the surface of a heart slice from the mouse ventricle reveals the fine structure of the parasympathetic innervation in the heart. Video 6 is a model simulating random movements (D_lat_ = 0.6 µm^2^ ⋅ s^−1^) of fluorescent molecules on the surface of a cubic cell (3 × 3 × 3 µm^3^), which has cylindrical protrusions (3 µm long; 0.5, 0.2, and 0.1 µm diameter).

## Results

### Single-molecule imaging in cultured cells and tissue slices

**Figure 3.**
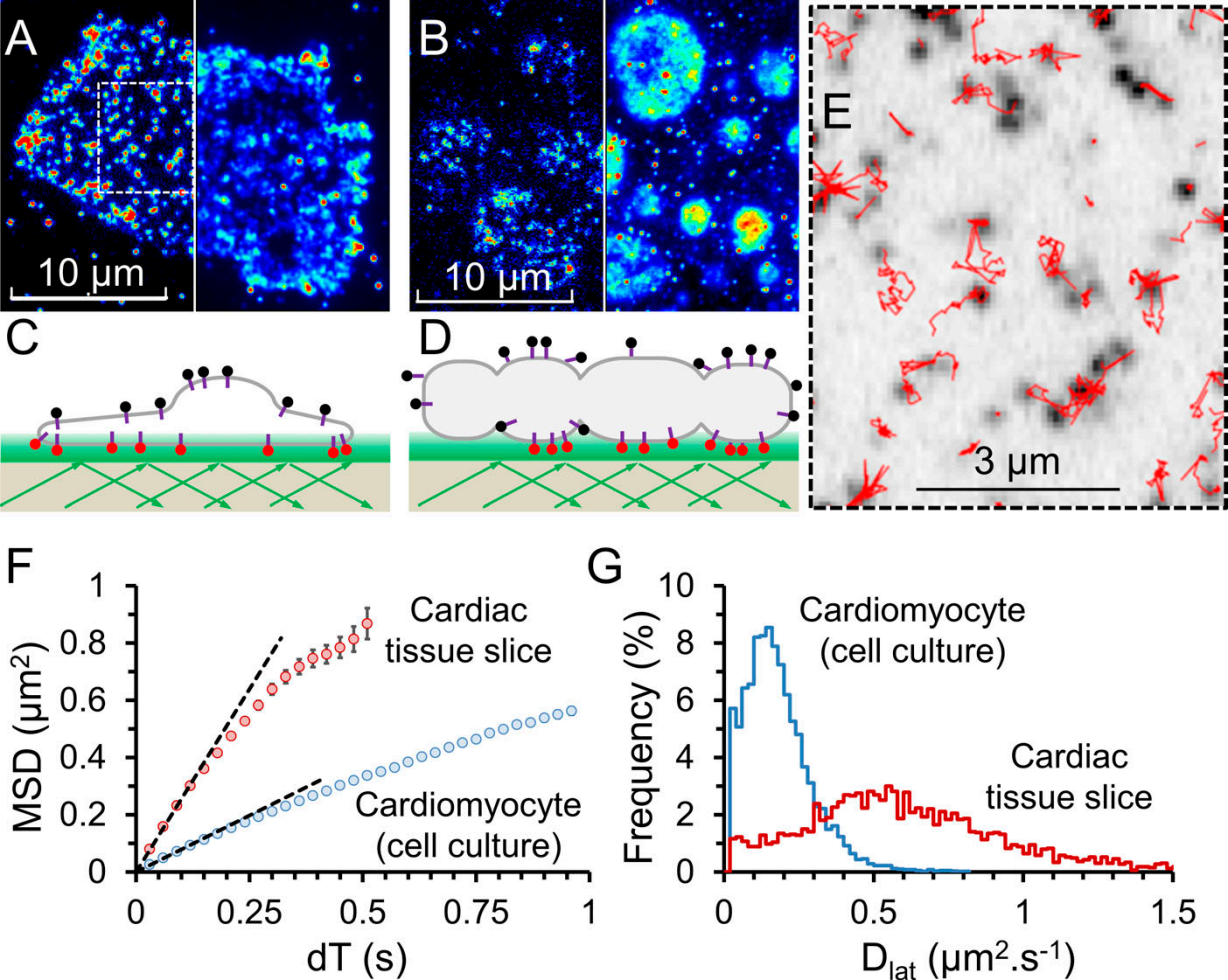
**Cy3B-telenzepine–labeled M_2_ muscarinic receptors on the plasma membrane of mouse cardiomyocytes.** **(A)** Left: A single video frame image of a primary-cultured cardiomyocyte, viewed by TIRFM. M_2_ receptors labeled with Cy3-telenzepine are visible as individual spots. Right: The same cell is shown as a normalized SD z-projection created from ∼500 video frames. **(B)** Left: A single video frame image of a small region of mouse cardiac tissue. Again, M_2_ receptors are visualized as individual spots of light. Right: The same cell is shown as a normalized SD z-projection from ∼500 video frames. **(C and D)** Cartoons depicting the TIRF imaging conditions in A and B.** (E)** Enlarged boxed region from A showing individual fluorophore trajectories (red traces). **(F)** The averaged MSD plotted against dT for M_2_ receptor movements in cardiac tissue slices compared with isolated cardiomyocytes. In this experiment, initial gradients are 2.56 and 0.79 µm^2^ ⋅ s^−1^, giving D_lat_ = 0.64 and 0.2 µm^2^ ⋅ s^−1^, respectively. See Table 1.** (G)** Histograms showing the distribution of D_lat_ estimates made for thousands of individual molecular trajectories measured from the two preparations.

An immediate observation made from our tissue-slice specimens is a significant difference between the shape of cultured, isolated cardiomyocytes and myocytes embedded in the heart tissue slice (Fig. 3, A and B; and Video 2). Primary cell culture requires cells to be dissociated from their native tissue before allowing them to settle, adhere, and regrow on a cell-culture plate. This process may select for adherent cells that have a large spread surface area and/or may cause cells to change morphology in order to adhere to the 2-D surface (Fig. 3 C). Cells in our tissue slices were allowed to maintain their native 3-D shape, and regions of the cell visible by TIRFM illumination had a smooth elliptical appearance (Fig. 3 D). Individual video frames of M_2_-expressing cardiomyocytes in a tissue slice do not correctly report the shape of the cell because the single molecules are relatively sparse. However, because the receptors are highly mobile, fluorophores soon explore the entire plasma membrane. When the normalized SD z-projection of the video frames is computed, cell shape then becomes apparent (Fig. 3, A and B, right). When an isolated cultured cell is strongly illuminated, photobleaching is most apparent in the central region of the cell because fresh (unbleached) molecules refill the membrane from the unilluminated apical surface (Fig. 3 A). This effect is greatly diminished in the tissue slices because the cell “footprint” is much smaller (Fig. 3 B), and because the M_2_ receptors move faster in tissue slices than in isolated myocytes. Together, this means that bleached and unbleached receptors equilibrate more rapidly and fluorescence appears more evenly distributed across the cell membrane. M_2_ receptors exhibited unrestricted diffusion, and fluorophore tracking (Fig. 3 E) showed MSD versus dT plots were linear (Fig. 3 F). Downward curvature at longer dTs can result from longer-duration trajectories being biased in favor of objects that move slowly for stochastic reasons because particle tracking works better, and they are over-represented in the datasets. We find the same curvature when we analyze simulated datasets of purely random walks.

**Video 2.** **Side-by-side comparison of receptor density and mobility in an isolated cardiomyocyte and in a heart slice from the ventricle of a mouse of the same age (0.5× real time).** An SD image, inserted at the end of the video, maps the cell borders.

Our data indicate movement of the M_2_ receptor is consistent with simple diffusive motion in an isotropic viscous medium (i.e., the plasma membrane). The average D_lat_ can be measured by least-squares linear regression to the initial slope of the MSD versus dT plot compiled from all molecular trajectories (Fig. 3 F). A histogram showing the distribution of D_lat_ estimates arising from analysis of each individual MSD versus dT molecular trajectory shows a broad, characteristic distribution (Fig. 3 G) best described by a γ function with median close to the initial slope of the population-averaged MSD versus dT plot. We repeated our experiments with mouse tissue slices at 37°C and found D_lat_ was two times faster compared with 23°C (Table 1).

**Table 1.** Comparison of M_2_ muscarinic G protein–coupled receptor diffusion (D_lat_) in mouse primary cardiomyocytes, mouse heart muscle slices (at 23°C and 37°C), zebrafish heart slices, and neuronal structures identified within the mouse heart slices

	Mouse cultured cardiomyocytes (23°C)	Mouse tissue slice (23°C)	Mouse tissue slice (37°C)	Mouse neuritis (23°C)	Zebrafish tissue slice (23°C)
D_lat_ ± SD (µm^2^ ⋅ s^−1^)	0.16 ± 0.04	0.62 ± 0.11	1.3 ± 0.2	0.4^a^	0.7 ± 0.06
Trajectories (n)	3,812	3,260	6,741	1,882	2,208
Cells (n)	8	17	11	N/A	11

N/A, not available

a The D_lat_ value stated here assumes free 2-D motion, whereby the gradient of the MSD-versus-dT plot is equal to 4D_lat_. Please see Discussion section for further explanation.

### Imaging heart tissue slices from adult zebrafish

**Figure 4.**
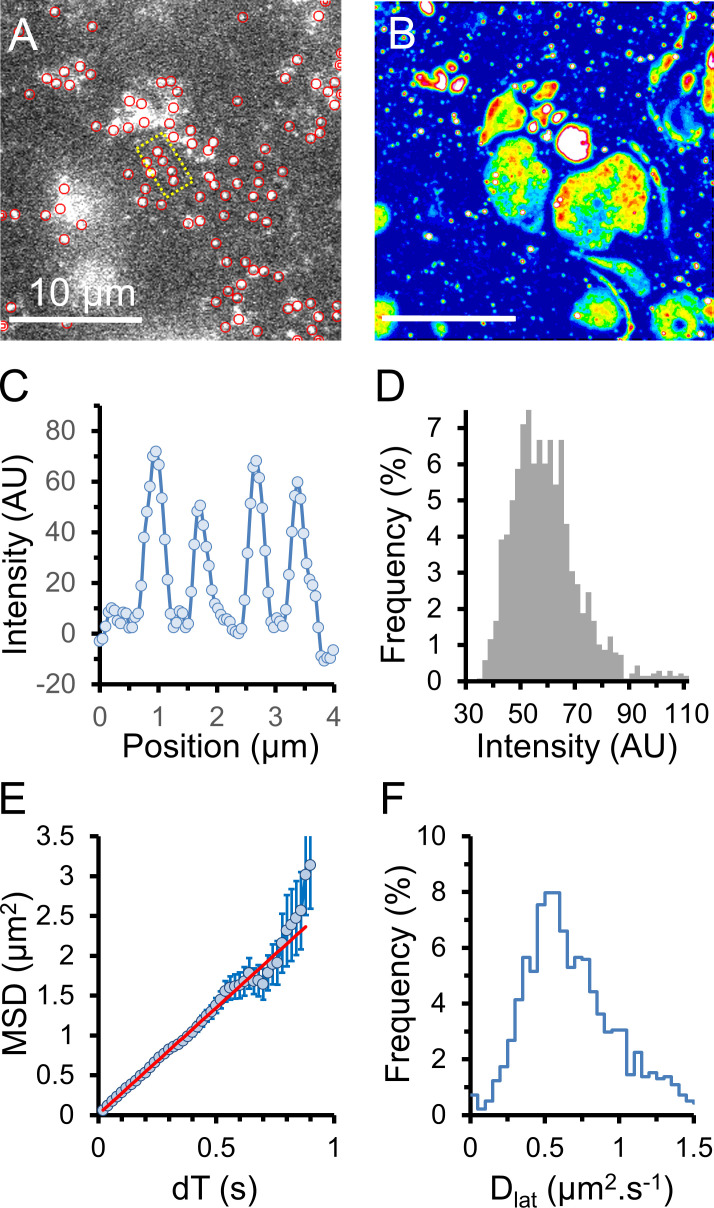
**Cy3B-telenzepine–labeled M_2_ muscarinic receptors on the plasma membrane of cardiomyocytes in an adult zebrafish heart slice.** **(A)** Single image from the beginning of the record shows individual receptors on the surface of the exposed cardiomyocytes. Note the difference in the M_2_ density between neighboring myocytes. **(B)** Normalized SD z-projection image created from the video record (∼500 frames) of the slice shown in A. The cell footprints are now clearly visible. **(C)** Line profile of image intensity within the yellow boxed area in A. Individual fluorescent spots have similar intensity (50–70 counts/pixel, above background, measured over a 5 × 5 pixel region). **(D)** Histogram showing the intensity distribution of fluorescent spots identified in the video recording (1,379 spots). Data are consistent with the M_2_ receptors being overwhelmingly monomeric. **(E)** MSD versus dT plot is fitted to a straight line (red) by least squares linear regression where the gradient = 4D_lat_, giving an estimated D_lat_ = 0.67 µm^2^ ⋅ s^−1^; error bars show SEM at each dT interval. **(F)** Histogram showing the distribution of D_lat_ estimates measured for every tracked object (*n* = 1,379); median D_lat_ = 0.7 µm^2^ ⋅ s^−1^. AU, arbitrary units.

The zebrafish, *Danio rerio*, is a model system for developmental studies, and we have compared its heart ultrastructure and membrane properties to those of mouse to establish single-molecule imaging across different model organisms. Zebrafish heart is known to express M_2_ muscarinic acetylcholine receptors (Hsieh and Liao, 2002), and we found the labeling procedure we developed for mouse cardiac tissue slices worked equally well with zebrafish heart (Fig. 4, A and B). We discovered that following the sectioning procedure, the heart retained its contractile properties (Video 3) as found for mouse heart slices, implying that it was fully functional and remained in its physiological state. After transfer to the relaxing medium, specimen movement was arrested, and video recordings could then be obtained without motional artifacts.

**Video 3.** **A slice of the zebrafish adult heart is still beating after 1 h labeling at 4°C.** The video was taken at 23°C.

The overall morphology of zebrafish heart slices appeared similar to that of mouse slices, and we found clusters of M_2_-expressing myocytes exposed at the slice surface. The density of M_2_ receptors was similar to those in mice (one or two receptors/µm^−2^), and individual fluorescent spots arising from Cy3B-telenzepine–labeled receptors showed unitary amplitudes (Fig. 4, C and D), while receptor mobility was slightly higher in zebrafish heart compared with mouse hearts (Fig. 4, E and F; and Table 1). As we found with mouse myocytes, MSD increased linearly with dT interval, which is consistent with a simple random walk produced by Brownian motion. In other words, we found rapid diffusive motion and no evidence for anomalous diffusive behavior in the cell membranes of our freshly extracted tissue samples.

### M_2_ receptors are mainly monomeric in both mouse and zebrafish

Analysis of the intensity distribution of individual fluorescent spots (Fig. 4, C and D) indicated that most of the receptors were monomeric, which is consistent with our earlier studies of mammalian cells (Hern et al., 2010; Nenasheva et al., 2013). This can be explained because when receptor density is low, dimer formation via collisional encounters is relatively rare compared with the dimer dissociation rate.

### Super-resolution images of mouse heart ultrastructure

**Figure 5.**
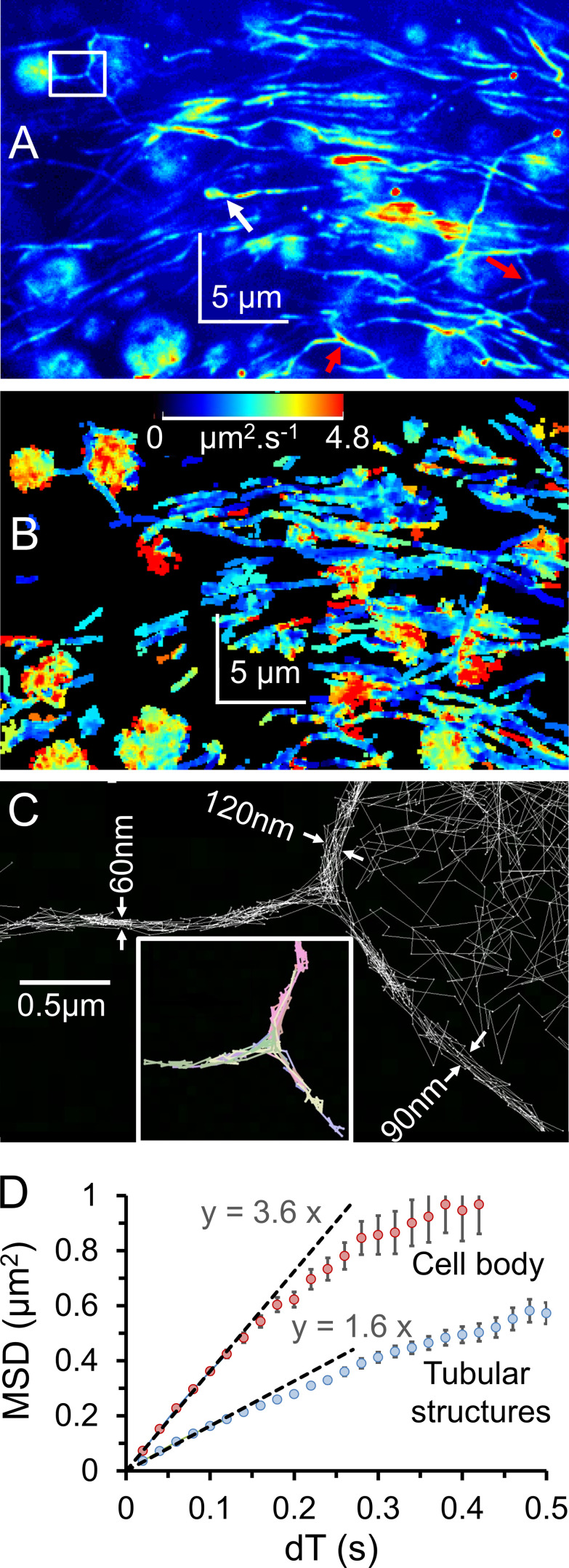
**Cy3B-telenzepine in vivo–labeled mouse heart slice.** **(A)** Normalized SD z-projection (shown using an arbitrary pseudo-color lookup table for display purposes) shows the distribution of rapidly moving M_2_ receptors on myocytes and nerve fibers. Nerve fiber “Y”-branches are marked by red arrows, and the white rectangle marks are the position of trajectories shown in C.** (B)** Color-coded average mobility map calculated using all single-molecule trajectories. The lookup table palette (top center) refers to the gradient of MSD versus dT plots ranging from 0 to 4.8 µm^2^ ⋅ s^−1^. Receptor mobility on nerve fibers (predominantly blue) appears slower than on cardiomyocytes. **(C)** Zoomed region from A shows individual molecular trajectories that delineate nerve ultrastructure with super-resolution. The lower insert shows selected colored trajectories of individual molecules that move in all three directions at the Y-branch point. **(D)** MSD versus dT plot indicates that receptor mobility is higher in the myocyte cell body than in the tubular neuronal structures (3.2 µm^2^ ⋅ s^−1^ and 1.6 µm^2^ ⋅ s^−1^ resp.; see Discussion for explanation).

We found that our simple matrix tissue-sectioning method preserved cardiac neuronal architecture and parasympathetic neurons, which express presynaptic muscarinic receptors (Jeck et al., 1988; Bognar et al., 1990; Oberhauser et al., 2001), which were labeled with the same fluorescent telenzepine analogue (Hern et al., 2010). This meant that both nerve and muscle cells were apparent in the same images (Video 4 and Video 5). Normalized SD z-projection images revealed nerve fibers terminating at M_2_-expressing myocytes (Fig. 5 A, white arrow). The tracking algorithm showed receptor density was lower in nerve axons (0.5–1.2 µm^−1^ axon length) compared with cardiomyocytes, and receptor mobility maps indicated lateral diffusion was slower in the axons (Fig. 5 B and Table 1), but see the Discussion section.

**Video 4.** **M_2_ receptor mobility in the adult zebrafish heart slice.** An SD image, inserted at the end of the video, maps the cell borders (0.5× real time). Bright sharp spots on the SD image represent Cy3B molecules on the coverslip surface. Their binding to coverslip, bleaching, or blinking creates big intensity fluctuations, which increase SD values.

**Video 5.** **Tracking individual muscarinic receptors on the surface of a heart slice from the mouse ventricle reveals the fine structure of the parasympathetic innervation in the heart.** The trajectory map is inserted at the end of the video to show all the trajectories detected in this record (0.5× real time).

It was impossible to visualize nerve ultrastructure from a single video image, but the stop-motion, super-resolution reconstruction approach (see Materials and methods) allowed nerve architecture to be visualized at unprecedented resolution (∼20 nm) in a live tissue slice (Fig. 5 C). There was a clear spatial distinction between receptor trajectories on the cardiomyocytes and those on nerve fibers; in other words, receptors (as would be expected) did not move between nerve and myocyte plasma membranes. The nerve fibers often appeared to branch (Fig. 5 A, red arrows) and exhibited Y-shaped junctions. At such junctions, receptors traveled back and forth along all possible paths, showing the plasma membrane was contiguous and therefore a single nerve-conduction path (Fig. 5 C and Video 5). Similar innervation patterns were also found in slices from zebrafish heart. We tracked fluorophore movement in cellular regions and in the extended tubular structures and found the gradient of the MSD-versus-dT plots for cellular regions was approximately twofold steeper than for the extended tubular structures (3.6 versus 1.6 µm^2^ ⋅ s^−1^, respectively; Fig. 5 D).

## Discussion

We have shown that single molecules can be observed at the plasma membrane of living cells from freshly explanted tissue slices. We used cardiac muscle as a model system and two different model organisms, mouse and zebrafish, to demonstrate how the technique can reveal information about protein–membrane interactions. We developed a number of novel single-molecule image-processing methods to explore our datasets, and this allowed us to investigate the molecular dynamics of a model G protein–coupled receptor (muscarinic acetylcholine receptors) within the plasma membrane of freshly extracted cardiac tissue slices.

Serial tissue sections were made using a custom-built matrix-sectioning device, and the cutting block cavity was readily modified to accommodate different-sized organ samples (mouse versus zebrafish hearts). In principle, the matrix block design could be adapted to section other organs, for example, skeletal muscle, liver, kidney, blood vessels, or brain. The imaging methods presented in this paper may be of particular value for studies of small model organisms (like zebrafish and perhaps *Drosophila melanogaster*), for which it is sometimes difficult to establish primary cell cultures due to poor cell survival rates or instances where the biological question demands the use of intact tissue.

We found that the lateral diffusion of M_2_ receptors in mouse heart slices from zebrafish was slightly faster than mouse (D_lat_ = 0.7 µm^2^ ⋅ s^−1^ versus 0.6 µm^2^ ⋅ s^−1^). However, imaging was conducted at 23°C for both preparations, which is 14°C lower than the body temperature of mouse and 5°C lower than for zebrafish (fish tank temperature, 28.5°C). We have shown here that M_2_ receptor mobility increases twofold for a 10°C increase in temperature in live tissue samples. This implies the D_lat_ of M_2_ receptors at physiological temperature for zebrafish and mouse might be more similar (D_lat_ ∼1.3 µm^2^ ⋅ s^−1^). In the future, it would be interesting to compare the mobility of membrane proteins in zebrafish acclimatized to different temperatures. There is evidence showing membrane viscosity is subject to homeostatic acclimation (Ernst et al., 2016), and many poikilothermic organisms are able to modify lipid composition and, thereby, the biophysical properties of their plasma membrane during acclimatization. The lateral mobility of M_2_ receptors in mouse tissue sections was significantly higher than in isolated, primary cultured cardiomyocytes, implying that adherent cell cultures may have altered membrane properties.

Over the past decade, a number of super-resolution imaging methods, like PALM and STORM (Rust et al., 2006), have been developed to reveal cell ultrastructure by high-precision, single-fluorophore localization and image reconstruction. To date, these approaches have mainly been applied to imaging the cytoskeleton in live cells and other structures in fixed specimens. Here, we have used a slightly different approach, localizing freely moving molecules in sequential video frames to create a super-resolution map of the membrane via stop-frame analysis (see Materials and methods). The mobility of transmembrane receptors (like the prototypical G protein–coupled receptors, studied here) reflects membrane viscosity and can be used as probes to give high-resolution maps of membrane biophysical properties.

**Figure 6.**
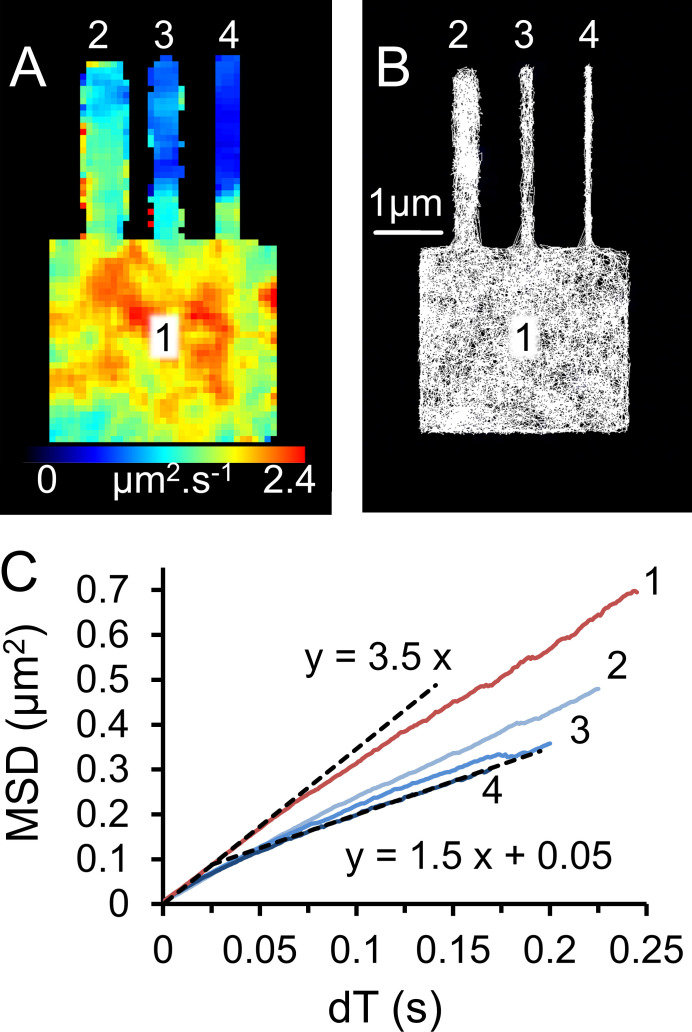
**Virtual cell model used to simulate video data and compare single-molecule mobility in different cell regions.** **(A)** Single fluorescent molecules were simulated, moving randomly (D_lat_ = 0.6 µm^2^ ⋅ s^−1^) on the surface of a cubic cell (3 × 3 × 3 µm^3^), which has a cylindrical protrusions 3 µm long. TIRFM imaging conditions, 50 fps, average fluorophore intensity 20 counts ⋅ pixel^−1^ ⋅ frame^−1^. The image shows the mobility map (initial slope of the MSD versus dT) for each of the tracked objects (*n* = 4,320) plotted on top of one another at the optical resolution limit of 250 nm (see Materials and methods). Note that the mobility is lower in the narrow cellular protrusions. **(B)** Super-resolution (∼10 nm) single-molecule trajectories form a meshwork of thin white lines. Notice that the diameters of the cellular protrusions are faithfully reproduced by the stop-motion, super-resolution tracking procedure (500 nm, 200 nm, and 100 nm). **(C)** MSD versus dT plots for different regions of the simulated cell. The numbers (1, 2, 3, 4) correspond to the cellular regions shown in A and B.

We found an apparent difference in membrane viscosity between cardiac and neuronal tissue (D_lat_ = 0.6 and 0.4 µm^2^ ⋅ s^−1^, respectively). However, this difference is most readily explained by the change in dimensionality of the diffusive motion. Our super-resolution stop-frame reconstruction revealed the diameter of the nerve fibers was ∼100 nm (which is close to EM measurements; Pauziene et al., 2016). The estimate of D_lat_ from the gradient of MSD versus dT plots is expected to differ by a factor of two (Berg, 1983) as the diffusive path changes from 2-D to 1-D. So, although we cannot totally exclude the notion that neuronal plasma membrane has a higher viscosity than muscle cells, the difference in D_lat_ that we measure is most easily consistent with the simple change in dimensionality of diffusive motion from a 2-D plane in the muscle cell to a virtually uni-dimensional line in the nerve axons. To test this idea further and also validate our analytical methods, we used a virtual cell model and generated a simulated single-molecule video (Mashanov, 2014). Fluorescent molecules were allowed to move randomly (D_lat_ = 0.6 µm^2^ ⋅ s^−1^), and the model geometry consisted of a “cell” (3 × 3 × 3 µm^3^) with narrow, closed, tubular protrusions of various diameters in order to mimic nerve fibers (Fig. 6). The simulations were made as realistic as possible using fluorophore intensity, photon noise, photo-bleaching rate, camera noise, and other background noise as close as possible to our experimental videos. We then analyzed the simulations (Video 6 and Fig. 6) using the analysis pipelines that we apply to our real experimental datasets. Our simulations show that we are able to satisfactorily measure the diameter of the very thin tubular structures and can accurately measure mobility of molecules moving in different regions of the cell. Importantly, our model recapitulates the finding of lower apparent mobility of membrane proteins moving within narrow tubular structures, so the apparent difference in muscarinic receptor mobility that we found for nerve and muscle membranes can actually be fully explained by the difference in their structural geometry.

**Video 6.** **Model simulating random movements (D_lat_ = 0.6 µm^2^ ⋅ s^−1^) of fluorescent molecules on the surface of a cubic cell (3 × 3 × 3 µm^3^), which has cylindrical protrusions (3 µm long; 0.5, 0.2, and 0.1 µm diameter).** TIRFM illumination mode. A trajectory map is inserted at the end of the video to show all the trajectories detected in this record, followed by “mobility map” (0.5× real time).

In previous studies, a combination of light microscopy and EM has shown the pattern of nerve fibers across the vertebrate heart (Thaemert, 1966; Pauziene et al., 2016). Fluorescence micrographs reveal nerve bundles, branching into individual fibers, while EM has been used to measure the cross-section of individual axons manually identified in the micrographs (Pauziene et al., 2016). To complement these histological studies, which rely on thin sectioning of fixed specimens, we have shown how live cardiac tissue slices can be used to show the contiguous path of nerve fibers across small regions of heart tissue, and this might assist studies of cardiac innervation pathways (Wetzel and Brown, 1985; Bognar et al., 1990).

### Conclusions

The results of our experiments show that the single-molecule imaging in ex vivo tissue slices provides a new level of understanding in membrane–protein interactions. The methodological approaches we have described will hopefully enable other studies of individual proteins in the lipid bilayer of live cells within their native multicellular tissue context.
